# A numerical study of a left ventricular expander for heart failure with preserved ejection fraction

**DOI:** 10.1098/rsos.230142

**Published:** 2023-07-19

**Authors:** Jonathan Weissmann, Yllan Charles Benoliel, Choon Hwai Yap, Gil Marom

**Affiliations:** ^1^ Department of Biomedical Engineering, Tel Aviv University, Tel Aviv, Israel; ^2^ School of Mechanical Engineering, Tel Aviv University, Tel Aviv, Israel; ^3^ Department of Bioengineering, Imperial College London, London, UK

**Keywords:** heart failure with preserved ejection fraction, medical assist device, numerical models, biomechanics, cardiovascular devices

## Abstract

Increased cardiac stiffness hinders proper left ventricular (LV) expansion, resulting in decreased volume and diastolic dysfunction. LV expanders are spring-like devices designed to improve diastolic function by facilitating mechanical outward expansion. Implantations in animals and humans have shown promising results, yet further evaluation is needed to assess a range of functions and the risk of use. In this computational study, the effectiveness and potential use of a generic LV expander were assessed by using previously generated finite-element models of induced heart failure with preserved ejection fraction (HFpEF). Following implantation, the treated models were compared to the corresponding untreated and healthy pre-induction models. The influence of device orientation and its material properties was also examined. Our results demonstrated a reduction in LV pressure and a volumetric improvement. Computed LV stresses have shown no gross irregularities. The device contributed to stress elevation during diastole while having a minor effect during systole, supporting a basic safety profile. This is the first study to use numerical analysis to assess LV expanders' performance on different HFpEF phenotypes. Improvement in heart function was demonstrated in both subjects, suggesting its potential use in various HFpEF manifestations, yet customization and optimal deployment are essential to improve heart performance.

## 1. Introduction

The incidence of heart failure with preserved ejection fraction (HFpEF) is rising and is considered a major contributor to morbidity and mortality worldwide [1], with a 2-year hospitalization rate of 35% and a 2-year mortality rate of 14% [2]. A hallmark of this disease is a high left ventricular (LV) filling pressure, while the ejection fraction (EF) is preserved above 50% [3]. Despite normal EF, the morbidity rate is high, and HFpEF patients commonly experience fatigue, dyspnea on exertion, and other signs and symptoms related to heart failure [4]. The high prevalence of HFpEF is associated with population ageing and various comorbidities [5–8].

Pharmacological therapies are mostly aimed at treating symptoms, and only a few have been shown to improve morbidity and mortality [9–11]. As a result, cardiac device therapies have been developed to improve cardiac function and ameliorate symptoms [12]. Pacemaker-like devices are intended to solve pathological electrophysiological activity, LV assist devices (LVAD) and other external pumps propel blood from the left ventricle to the rest of the body [13], and interatrial shunts reduce left atrial pressure [14]. LV expanders, such as the CorAssist CORolla device (CorAssist Inc., Haifa, Israel), were designed to improve cardiac relaxation performance via outward expansion of the left ventricle. The CORolla is a spring-like expander, implanted transapically, that applies outward force to the ventricular wall to improve its filling. A simplified electrical analogy model demonstrated that this device can transfer energy from systole to diastole due to its recoil properties, and it has been successfully validated clinically [15]. It was primarily tested on mature animal models [16,17] to evaluate complications after implantation and examine the short-term (up to six months) impact on the systolic phase. Only a select few patients underwent implantation in a first-in-human trial [12], which mainly focused on long-term anatomical and physiological assessments using echocardiogram and functional tests. One year after the implantation, an improvement in LV mass index was observed, while functional improvement was already evident within six months. Despite these encouraging findings, the effectiveness of the expander in hearts with varying degrees of hypertrophy remains unexplored. Further evaluation is needed to assess its range of function among patients and the risk of its use [15].

Cardiac *in silico* modelling can provide a means to evaluate device safety and offer a better mechanical understanding through the calculation and quantification [18] of vital information [19]. Cardiac *in silico* techniques may not account for extracardiac phenomena, and challenges still remain in modelling spatially varying material and cardiac dynamic behaviour. Yet, these techniques offer the opportunity to test several anatomies and device configurations, as well as investigate the effect of physiological alterations, for example, in haemodynamics and contractility, while overcoming *in vivo* experimental challenges [20]. In the last few years, models have been developed to test the interaction between cardiac implanted devices and the beating heart [21–24]. These studies primarily involved the evaluation of various stents made from different materials and devices aimed at valvular diseases. Their objective was to quantitatively assess the cardiac function after implantation, estimate the risks of clinical complications, such as blood flow obstruction, and evaluate the long-term durability of the devices. A few *in silico* models have investigated medical devices in HFpEF. The simulations have been conducted to model flow pumps that are designed to restore cardiac output and normalize left arterial pressures by driving blood from the left atrium to the aorta. Simulations of both continuous [25] and pulsatile flow pumps [26,27] have been performed to assess the impact of circulatory devices in HFpEF and optimize their design. Furthermore, advanced LVAD models that use realistic geometries [28–30] have also been developed to study their effect on ventricular pressure. Granegger *et al*. [31] have recently used lumped parameter models to compare several device-based methodologies for HFpEF, including various pacemaker-like devices and an interatrial shunt. However, due to the hemodynamic complexity and high phenotypic variability, personal selection of device-based therapy was impractical. Relatively few studies have tested the use of spring expanders [15,32], yet due to the complexities involved, only a simplified electrical-analogue lumped model was developed to explore their basic use [16].

In this HFpEF computational study, we sought to simulate the implantation process of a spring-like expander, such as the CORolla, in subject-specific HFpEF models with the aim of evaluating its impact on LV function during the cardiac cycle and exploring its ability to transfer energy to diastole in a credible *in silico* models. Basic alterations in device orientation and stiffness were introduced in subsequent analyses to improve device performance. Our study is the first to explore the physiological and mechanical responses to the treatment based on subject-specific finite-element (FE) analyses. In this study, post-implantation computational models were compared to the corresponding generated healthy and pre-implantation models, representing the porcine clinical status before and after disease induction, respectively [33]. In addition, several device configurations were examined. We were, therefore, able to provide a high-fidelity assessment of device effectiveness and, more importantly, assess possible directions to optimize its potential use.

## 2. Methods

### 2.1. Device modelling

**Figure 1.**
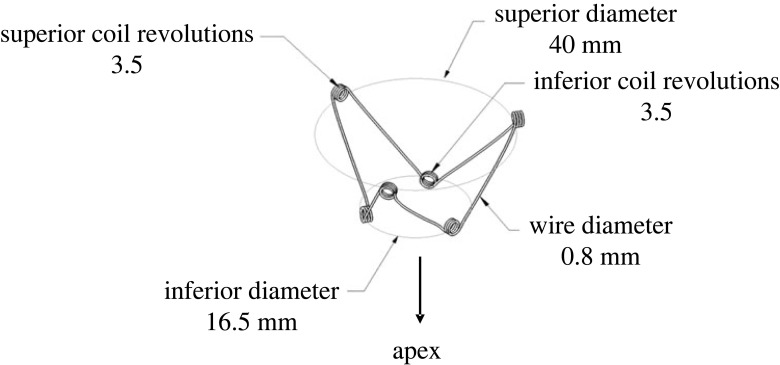
The structure of the generic expander's metallic frame and its dimensions.

A generic spring-like expander device that resembles the CORolla was modelled with dimensions that fit the subject-specific anatomies. The device comprised six elastic wires with six coils between them that create three ‘arms'. An illustration of the device and its components, as well as the corresponding dimensions, is shown in figure 1. The coils act as torsion springs that store energy during heart contraction. The apical coils are connected to three thin elastic polyester threads (not shown in figure 1) that intersect in the symmetry axis, where an additional fixation suture anchors the device to the apex [15].

**Figure 2.**
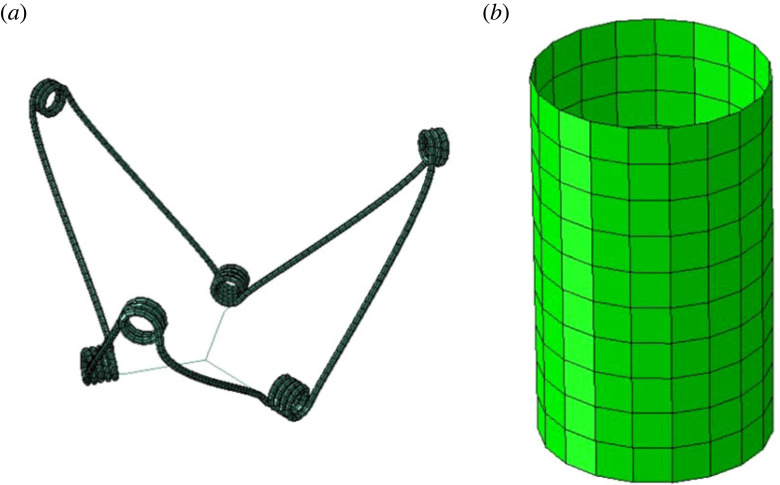
A view of the meshed expander device model (*a*) and the mesh of the cylindrical sheath (*b*, not to scale).

The expander geometry was drawn in Solidworks (Dassault Systèmes) using a parametric approach and underwent further processing in SpaceClaim software (ANSYS Inc.) to produce optimal line tangencies of the arms. The geometry was imported into Abaqus (Simulia, Dassault Systèmes), where it was meshed, and the threads were created. The metallic frame comprised 2652 three-dimensional eight-node elements (C3D8R), while the elastic threads were made of 24 truss elements (T3D2), assuming they can only be in tension (figure 2).

The metallic frame is made of a cobalt–chromium alloy [15], which is frequently used in metal implants for many medical applications, predominantly in cases where strength and wear resistance are required [34]. The MP35N alloy composition was chosen and was defined as homogeneous, isotropic and elastic linear, with a Young's modulus (*E*) of 232.8 GPa, Poisson's ratio (*ν*) of 0.3. The thin elastic threads, made from polyethylene terephthalate material as in surgical sutures, were defined as having linear elastic properties (density = 1000 kg m^−3^, *E* = 1.2 MPa, *ν* = 0.49) [35].

A cylindrical sheath was modelled to represent both a crimper for folding the device and a delivery system for the subsequent deployment. It was constructed in Abaqus and meshed with 200 four-node shell elements (S4R) of 0.1 mm thickness (figure 2). The 65 mm-diameter cylinder was assumed to be much stiffer than the device (steel-like material), and it was positioned concentrically with the device.

### 2.2. HFpEF porcine heart model

The HFpEF heart models in the current study are based on our previous FE heart models [33] and pertain to porcine subjects after the induction of HFpEF [36,37]. These subjects demonstrated key anatomical and physiological characteristics of the disease, including moderate hypertrophy, LV mass growth, high pressures, volume reduction and preserved ejection fraction (table 1). The corresponding healthy configurations used in this study for evaluation were reconstructed from cardiac magnetic resonance imaging (cMRI) scans and pressures data, taken before the induction process. For all scenarios, corresponding FE cardiac models were developed by morphing the mesh of the Living Heart Porcine model (LHPM) [38,39], while retaining mesh topology and scaled material properties, to represent a healthy tissue in the healthy models and myocardial stiffening in the HFpEF models [33,40].

**Table 1.** Anatomical and physiological properties of the HFpEF FE model.

	end-systole		end-diastole		stroke volume (ml)	ejection fraction (%)	LV mass (g)	LV mass index (g m^−2^)
	volume (ml)	pressure (mmHg)	volume (ml)	pressure (mmHg)				
Subject 1	20	92	51	13	31	61	132	136
Subject 2	22	86	56	20	34	61	122	120

**Figure 3.**
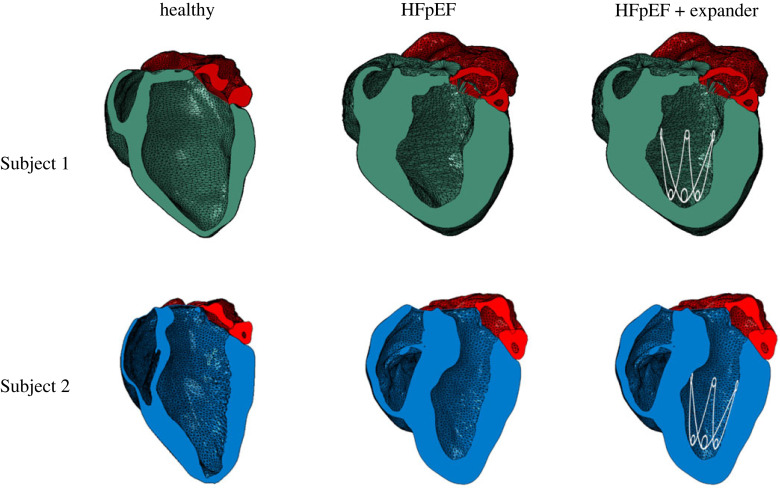
The healthy and HFpEF FE models of both subjects (left and middle columns), presented in a long-axis view, where the ventricles are in blue and green and the atria in red. Illustration of the same HFpEF model with a schematic description of the expander shows the desired implantation location. Adapted from Weissmann *et al*. [33] (published under CC-BY licence).

The FE HFpEF models were chosen to simulate device implantation and to assess its influence on heart function at different levels of hypertrophy. Additional FE models of these same subjects, prior to HFpEF inductions, allowed further comparison of the treated hearts to both their pathological and healthy states. The four heart models are shown in figure 3, along with schematic of two additional models of hearts with the devices.

### 2.3. Device implantation and unsheathing

**Figure 4.**
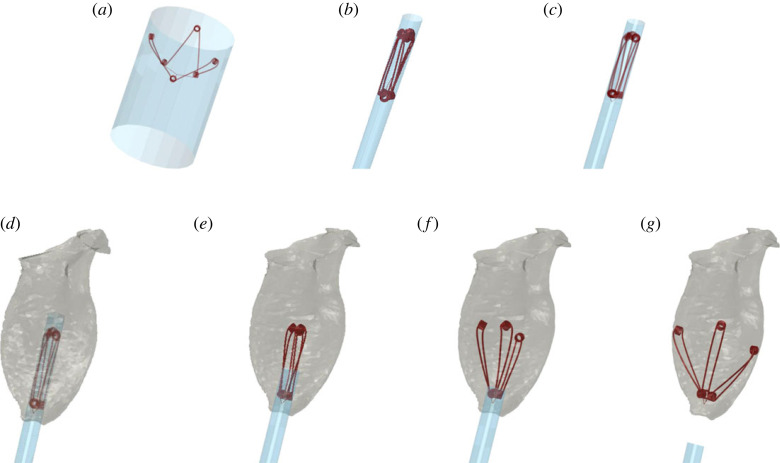
The deployment modelling of the expander device included its crimping (*a*,*b*) and thread pulling (*c*), followed by its positioning in the left ventricle (*d*). The sheath was gradually retracted (*e*,*f*), and finally the thread joint was anchored to the apex (*g*).

As an initial step in a stand-alone analysis, the device was crimped (figure 4*a*). Gradual radial displacements were defined for the cylindrical crimper's nodes to reduce its diameter to 20 mm (figure 4*b*), which concomitantly transferred contact forces to the device. The threads of the device were pulled outwards (figure 4*c*), via displacement boundary conditions, to avoid entanglement and winding around the lower coils. The position of the cylindrical sheath and the device, as well as residual strains, were extracted and imported into the subsequent implantation analysis.

The device implantation was performed in both models and included several steps, in accordance with the detailed description by Feld *et al*. [15]. In short, implantation is performed transapically. The CORolla device is inserted via the delivery system and guided to the appropriate orientation. The sheath of the delivery system is pulled backward to allow the device to be released. Once in place, the device is anchored to the apex with a fixation suture.

In clinical practice, medications are administered to regulate heart rate and allow controlled deployment, minimizing potential obstacles posed by contractions. This helps to mitigate technical challenges, maintain proper positioning and reduce the likelihood of complications [16]. Therefore, in the FE analysis, the device and the sheath in their folded configurations were positioned in the left ventricle at peak diastole (figure 4*d*), with no contraction during the deployment process. The elastic thread intersection was placed adjacent to the apex, and the arms were aligned with the long axis of the left ventricle. Retraction of the sheath (figure 4*e,f*) was modelled by a gradual outward displacement of the sheath, by applying displacement boundary conditions to each node of the sheath. The boundary conditions included intermediate pauses during unsheathing to reduce excessive movement of the device and allow for its recoil stabilization. The thread joint was anchored to a node in the apex using linear constraint equations (figure 4*g*). Device implantation in each of the porcine subjects is shown in electronic supplementary material, video S1.

### 2.4. Cardiac cycle models and device efficiency assessment

**Figure 5.**
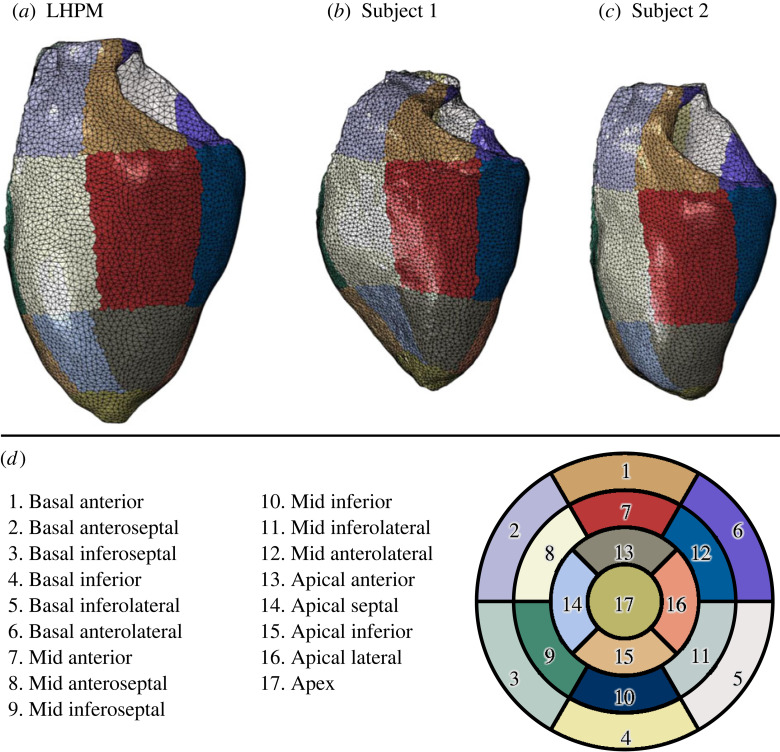
The segmented mesh for the LHPM (*a*), Subject 1 (*b*) and 2 (*c*) according to the standard myocardial segmentation and nomenclature (*d*) for tomographic imaging of the heart [41].

The impact of the device on the systolic and diastolic functions was assessed by computing full cardiac cycles of the HFpEF models with the implanted device in them. Similar to the previous analyses of the healthy and HFpEF models, six cycles were modelled to ensure numerical convergence and a temporally cyclic solution. For each model, a pressure–volume (PV) curve of the left ventricle was plotted, and the treatment was compared to the corresponding pre-induction healthy condition as well as to the HFpEF conditions without the implanted device. To measure the local and global effects of the device on the left ventricle, stress distribution was also calculated for each HFpEF scenario, both with and without the implanted device. The corresponding healthy models, and their different cross-sectional areas and material properties, cannot be used for stress comparison. In the pre- and post-implantation configurations, for each element across the myocardial wall of the left ventricle, von Mises stresses were obtained at 200 equally spaced time instances from the last cardiac cycle. Volume-weighted average stress was calculated as a function of time to evaluate differences between the treated and untreated models for each porcine subject. Since the exerted forces on the myocardium aid the diastolic expansion, the spatial stress distribution during the diastolic phase was compared. Using an in-house Matlab code, the left ventricle was divided into 17 segments, as recommended by the AHA [41] (figure 5), and a volume-weighted average stress was calculated for each segment, which was then temporally averaged during diastole. Given the identical topology in all generated FE models, segmental stress between the treated and untreated HFpEF models could be compared. This comparison can help in examining the device's role in absorbing energy from the systolic contraction and transferring it to the diastolic phase. Finally, the strain energy in the device throughout the cycle was computed.

### 2.5. Device orientation and its mechanical properties

**Figure 6.**
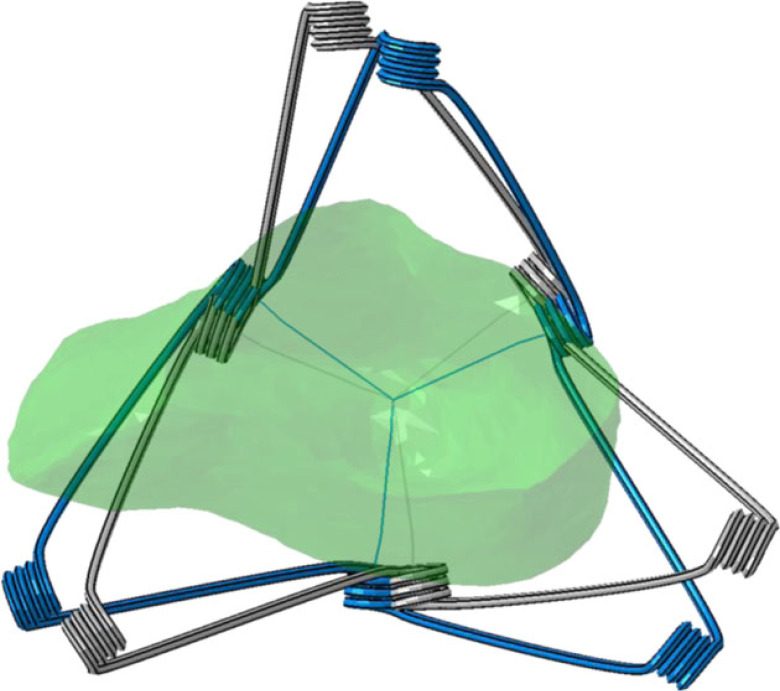
Schematic view of the device before crimping in their relative orientations of the first simulation (blue) and after rotation around the long axis of the heart (grey). The endocardium of the left ventricle is shown in green.

The impact of device orientation on the cardiac cycle was tested next. For this proof-of-concept analysis, the first subject was chosen for device implantation since there were salient differences in volume and pressure between its healthy and HFpEF configurations. The device was slightly rotated (approx. 11°) around the long axis of the heart to change the orientation of the ‘arms', ensuring that their opening is directed against the LV wall in a different location (figure 6). Note that the long axis of the heart does not coincide with the central axis of the device's delivery system. The device dimensions, apex fixation point, and initial stress were not altered. Finally, additional simulation in the new orientation was carried out, using a slightly more flexible cobalt–chromium alloy, with *E* = 220 GPa. Full cardiac cycles were computed for all scenarios. Ventricular displacements of the diastole relative to the systole were compared between the cases, in addition to the previously mentioned quantitative comparisons, such as PV curves.

## 3. Results

### 3.1. Effect of the expander device on the cardiac cycle

**Figure 7.**
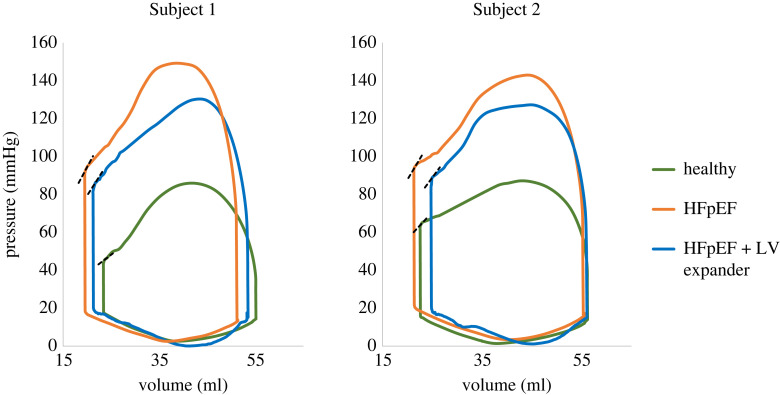
The pressure–volume curves for each porcine subject. The HFpEF configuration with the implanted device is denoted in blue, and the healthy and pre-implantation HFpEF configurations are denoted in green and orange, respectively. The dashed black lines denote the ESPVR slope curve corresponding to each scenario.

The last cardiac cycle, which was fully converged and cyclic [33], was used for the comparison of the PV loops in the six models. Figure 7 compares the PV loops of the treated HFpEF (with the device), the pre-implantation HFpEF condition, and the corresponding healthy condition prior to disease induction [33]. HFpEF is associated with an elevation in pressure, to maintain EF within normal range [5]. An overall unfavourable moderate reduction in pressure was observed after implantation in both subjects, with a decrease of 13% and 11% in maximal pressure, for Subjects 1 and 2, respectively. The pressure during diastole remained below 20 mmHg, within the physiological range.

The end diastolic volume (EDV) and the end-systolic volume (ESV) increased in both models. In Subject 1, the ESV after treatment was between the ESV values of the HFpEF and the healthy configurations, while in Subject 2 it was larger than both. The EDV, however, was equal to or lower than the healthy EDV value, which reflects ideal values, computed in both cases. The stroke volume (SV) in the treated Subject 1 model was 32.3 ml, compared to 32.2 ml and 31.2 ml for the corresponding healthy and HFpEF conditions, respectively. Accordingly, in Subject 2, the SVs were 31.4 ml versus 35.4 ml and 34.0 ml, respectively. In both cases, a preserved EF was maintained above 50%, with 60% and 56% for Subjects 1 and 2, respectively. A mild reduction to 52% was observed in Case 2 after the expander implantation.

End-systolic pressure–volume relationship (ESPVR) is a measure of cardiac contractility, which may also indicate differences between healthy and HFpEF conditions. The sloped lines (dashed black lines) in figure 4 represent this pressure to volume ratio at the end of the systole and show that after treatment, the ESPVR was reduced relative to HFpEF and brought closer to the healthy conditions (table 2).

**Table 2.** Calculated ESPVR for the healthy, HFpEF and HFpEF with LV expander conditions of each porcine subject. Values are in mmHg ml^−1^.

	HFpEF	HFpEF with LV expander	healthy
Subject 1	4.7	4.0	1.9
Subject 2	4.4	3.6	2.8

### 3.2. Myocardial stress and device energy assessment

**Figure 8.**
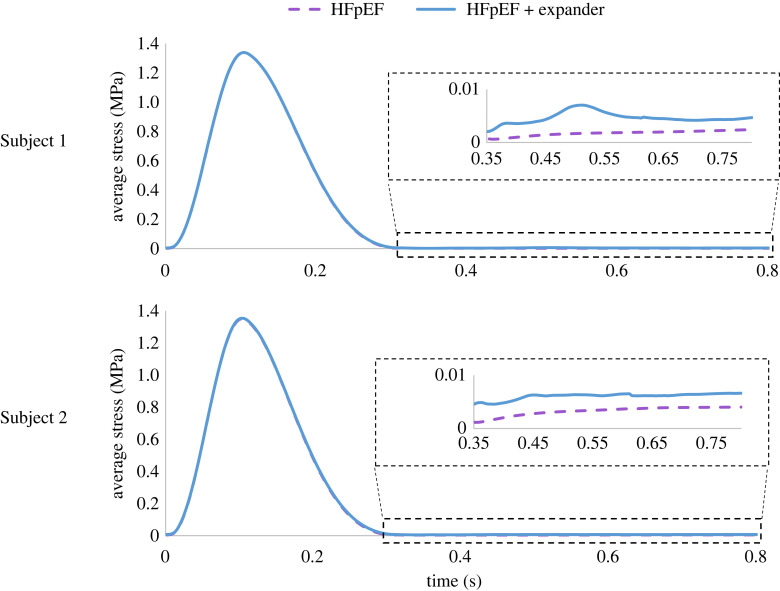
Average LV stress as a function of time during the heart cycle in the two porcine subjects. Comparison between the treated (light blue) and untreated (purple) configurations with zoomed-in panels of the diastolic phase.

The curves in figure 8 depict the average stress in the left ventricle as a function of time for all HFpEF scenarios. The material properties differ between the two subjects yet are identical for each scenario of the same subject, representing HFpEF conditions before (dashed purple) and after (light blue) device implantation. The computed stress range was less than 0.01 MPa during diastole and less than 1.4 MPa during systole for both subjects. In comparison to their pre-implantation settings, both devices facilitated an increase in diastolic pressures while having limited influence on the systolic average stress.

**Figure 9.**
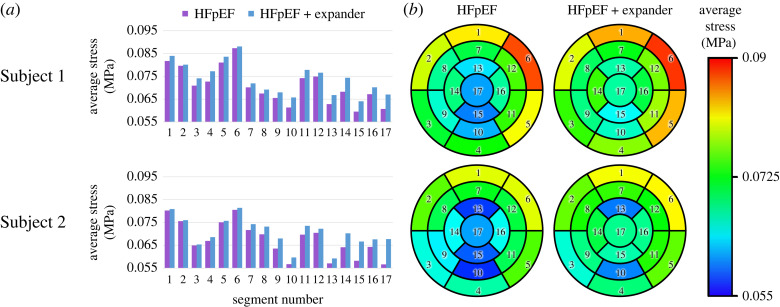
Time-averaged diastolic stress distribution per LV segment (figure 5). Left: Comparison between the treated (light blue) and untreated (purple) configurations. Right: Bullseye spatial representations of the LV coloured according to the magnitude of the average stress.

The average diastolic pressure distribution across the LV segments is illustrated in figure 9. The column charts compare the treated and untreated configurations for each porcine subject. The average von Mises stress has increased in both implanted device configurations when compared with the matching untreated HFpEF model. The major changes can be seen in segments where the expander coils and the LV wall were in immediate contact (14, 17 for Subject 1 and 15, 17 for Subject 2). Bullseye heatmaps on the right side of figure 9 provide a topographic representation of the left ventricle (figure 5) to demonstrate schematic spatial differences in normalized average stress for each scenario. Although the average LV stress was largely similar between the two subjects (figure 8), the distribution across the segments differed substantially. The regions most affected were the apex and its neighbouring segments, where the expander is fixated, and the LV cavity is relatively narrow.

**Figure 10.**
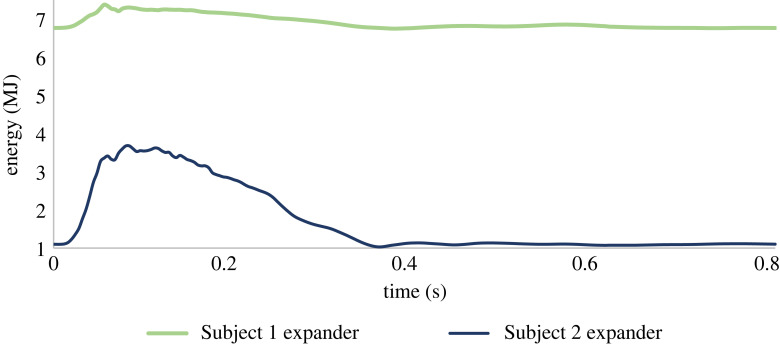
LV expander energy throughout a single cardiac cycle for each porcine subject.

**Figure 11.**
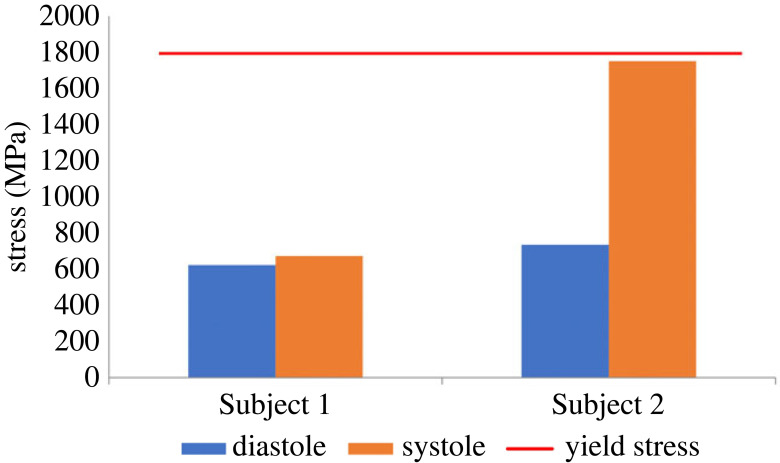
Von-Mises stress at peak systole (orange) and peak diastole (blue) for each porcine subject. The red line denotes the cobalt–chromium yield stress.

A strain energy as a function of time curve was plotted for each of the devices and presented in figure 10. In both models, periodic behaviour was observed, where energy is stored during systole and released during diastole, as demonstrated by a sharp increase during systole. The calculated energy in Subject 1 was higher (7 MJ versus 2.5 MJ, for Subjects 1 and 2, respectively), but its amplitude was lower. It is also possible to look at the working conditions of the device in terms of stress. Von Mises stresses in the LV expander during systole and diastole were calculated for the two subjects (figure 11). Despite a significant increase in stress at systole in Subject 2, which fits the large energy amplitude in figure 10, the stress did not reach the yield strength of the cobalt–chromium MP35N alloy (1790 MPa) [42] and stayed in the linear range.

### 3.3. Effect of device orientation and its material properties

**Figure 12.**
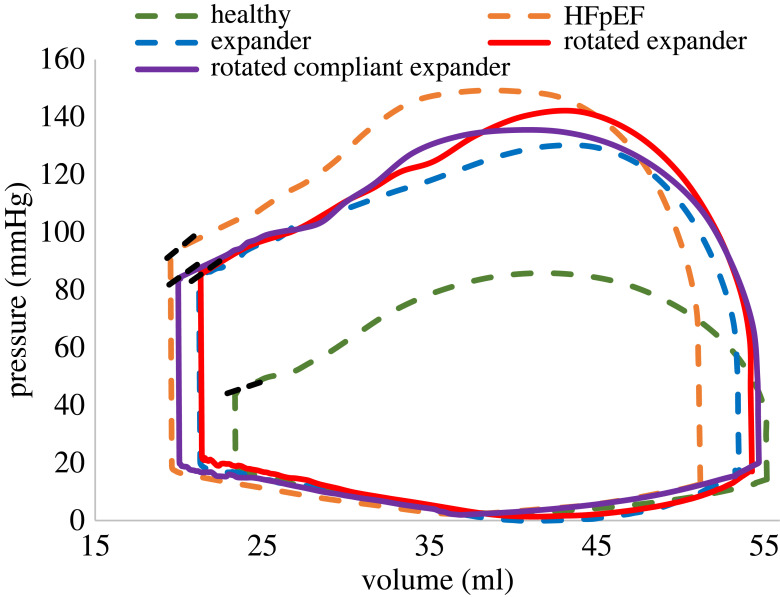
The pressure–volume curves for Subject 1. The green and orange dashed lines represent healthy and HFpEF configurations, respectively, and the original implantation is denoted by the dashed-blue line. The purple and red curves denote the LV expander after device rotation and after rotation and material stiffness reduction, respectively. The dashed black lines denote the ESPVR slope curve corresponding with each scenario.

For each device configuration, a pressure–volume curve was plotted as shown in figure 12. Rotation of the device (red curve) contributed to the enlargement of the EDV while mildly decreasing the LV pressure relative to the disease (dashed-orange curve). A maximal increase in EDV was observed following device stiffness reduction, where the diastolic volume was similar to the healthy (dashed-green) pre-induction condition (approx. 55 ml). The rotation did not influence the ESV (red versus dashed-blue curves). However, the more compliant expander allowed the heart to contract and reach a volume closer to the pre-implantation of the device (approx. 20 ml). The ESVPR values had been mildly affected by the rotation and alteration of the device material properties. The SV following device rotation was 33.2 ml, compared to 32.3 after the initial implantation. The use of a more compliant material has further increased the SV to 34.7 ml. In all scenarios, the EFs were more than 60%.

**Figure 13.**
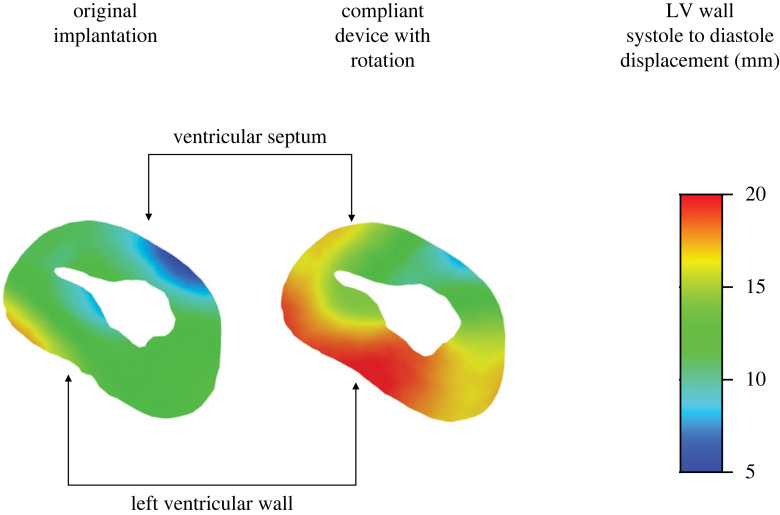
Comparison of systole-to-diastole displacement distribution in the LV wall in the original implantation (left) and after its rotation and material stiffness reduction (right). The contours are on a short-axis cross-section at peak diastole.

The LV wall displacement was calculated to quantify the effect of the rotated compliant device relative to the original orientation of the implanted device. In figure 13, relative wall displacements in a short-axis view are shown, where the height was chosen according to the superior coils' location. This relative displacement is defined as the local displacement between the systolic and diastolic configurations of the last cardiac cycle. As expected from figure 12, the rotation and stiffness of the device have contributed to an outward expansion of the LV wall. It is demonstrated by both the displacement contours and the overall LV inner cavity area (in white). Although the more compliant device undergoes larger displacements during the cycle, the calculated stresses at peak diastole and systole (591.6 MPa and 810.3 MPa, respectively) were both below the cobalt–chromium MP35N alloy yield threshold (1790 MPa).

## 4. Discussion

Two numerical models of HFpEF induced swine were used in this study to investigate the therapeutic effectiveness and essential risk factors of an expander device similar to the CORolla device. To determine treatment potential, two models were tested and compared with the corresponding healthy reference configuration, which was generated based on the same subject prior to HFpEF induction [33]. For Subject 1, possible improvements to the implantation outcomes were explored by considering slight rotation and material property alteration.

The device had no effect on overall systolic and diastolic behaviour, as evidenced by similar shapes of PV curves with no conspicuous extensive interferences. Nonetheless, pressure and volume were altered in both models. As expected, HFpEF models are characterized by elevated LV pressure. Our results have shown that the device has successfully helped reduce LV pressures in both models by more than 10% (figure 7). The resultant pressures are still higher than in the healthy states. However, it is likely that this pressure reduction is undesired. Hypertrophy is a compensation mechanism to preserve heart function [43]. In our particular study, the porcine models that underwent HFpEF induction via aortic cuffing had high LV pressures, which were vital for their survival. A significant reduction may therefore compromise this mechanism and impair heart function. In our subsequent models, we sought to minimize this systolic pressure reduction.

The healthy and the HFpEF configurations differ from one porcine model to the other. The elementary volumetric differences are mainly due to material characteristics, the degree of hypertrophy, and the direction of the muscle fibres. Nonetheless, positive trends in increasing the EDV have been observed in both porcine models following device implantation. In Subject 2, with the implanted expander, the volume has mildly increased and matched the computed value in the corresponding healthy configuration, while in Subject 1 the change was more salient, yet the computed EDV was smaller than in the healthy condition (figure 7). These EDV results insinuate that the treatment in Subject 1 has not reached its full potential, prompting the decision to test potential improvements only on this subject. The ESV has increased in both models and was not limited by the original healthy ESV value.

The device also affects additional clinically important measures that can be obtained from the PV loops. The alterations in EDV and ESV have inevitably affected the SV. In Subject 1, the SV has slightly increased, whereas in Subject 2, it has diminished, probably since the device had little effect on the EDV, which was close to the original healthy values. The EF has changed accordingly, yet remained within preserved values, demonstrating the device's safety profile. The ESPVR measure was improved in both subjects but did not reach healthy values (table 2).

The pressure and volume results, as well as the clinical measures (SV, EF and ESPVR), all indicate that the device can improve the pathologic conditions, but that there is still potential room for improvements. Such improvements can be achieved via careful device design and its adaptation to a specific anatomy. However, to evaluate device potential, generic device models were used, which were neither optimized nor fully based on the CORolla design.

The CORolla is a spring-like device that is intended to transform energy from the systolic phase to the diastolic one [15]. While previous attempts to model this effect [16] only considered the device's global function and assumed the pressure could be a proxy for energy, our detailed model allowed us to investigate the temporal and spatial stress and displacement distributions as well as the energy in the expander device. Our analysis has shown that the device has led to an increase in stress during diastole while having a minor effect on stress magnitude during systole (figure 8). This can be explained by the very high stresses that the heart muscle is exposed to during systole because of the contraction itself. The similar results in the very different pathologies of Subjects 1 and 2 imply that the device's function is not sensitive to the specific disease, an obviously desired trait of a medical device. Albeit the similar average LV stress of the two subjects, the stress distribution among the segments is different (figure 9). The device had a mild impact on the distribution itself, as it affected all segments. Nevertheless, the most afflicted regions were in contact with the device coils. An optimal deployment of the device and tailoring its dimensions to a specific anatomy is therefore important to reduce unnecessary elevation in LV stress.

In both subjects, the maximal calculated stress was less than the yield strength (figure 11), further supporting our assumption that the material is within the elastic linear range. The energy of the installed devices was calculated throughout the cardiac cycle (figure 10) and has demonstrated, as expected, the ability of the spring device to absorb energy during systole and release it in diastole. Interestingly, despite having divergent energy profiles with high baseline magnitudes in Subject 1 versus high amplitude in Subject 2, both have successfully improved cardiac performance. These distinctions can be attributed to the geometry and pathologic tissue properties of the two subjects [33]. Subject 1 had a stiffer myocardium with smaller volumes, which encumbered the device and led to a higher baseline computed energy and lower amplitude. Still, as mentioned before, the fact the device was still able to improve the conditions of the two distinctive pathologies demonstrates its potential to improve heart function in various manifestations of the HFpEF disease.

As demonstrated in the concluding analyses, the orientation of the device may affect heart performance, suggesting that the LV expander should be deployed and positioned with careful consideration. In comparison with the first implantation in Subject 1, the PV curves following device rotation (figure 12) have shown a milder reduction in pressure, as desired in our case. It also enabled a better LV expansion, thus improving the SV and EF. The additional modification of device material has allowed for better contraction of the LV. The less stiff device interfered less with heart contraction, allowing the ventricle to almost reach its systolic volume without the device. Obviously, this volume is much smaller than in the healthy state as a result of the muscular hypertrophy. Reaching smaller volumes is not only desirable because it increases the SV, but it also enables better energy absorption by the expander because it undergoes larger compression during systole. Therefore, the increased energy absorption itself helps in the energy transfer to the diastole, as evident by the better expansion of the device (figure 13). This combination of increased EDV and reduced ESV directly increases SV, making this scenario the one with the largest SV in our comparison. Still, the considered modifications were mild (approx. 11° in orientation, approx. 5% in stiffness) and are intended only to demonstrate potential directions to optimize the device design and adapt its implantation to a specific anatomy. Moreover, it demonstrates the potential of such numerical methods in the optimization process.

This study focused on the effect of an expander device on heart function. We sought to perform a fundamental assessment of device function on different degrees of HFpEF. Investigation of the cardiac impact on the device and fatigue analysis are beyond the scope of this paper. We assumed that a single device design could treat both subjects because we did not attempt to investigate device optimization. The diameters, coil revolutions and angles can be altered to tailor the device to a specific anatomy. A dedicated subject-specific investigation of several devices of various dimensions, via parametric analyses, could provide more insight towards optimal design by evaluating the individual effect of each component. In our analysis, only cobalt–chromium alloys were considered, with merely a mild stiffness difference that can be controlled in the manufacturing process. Other biocompatible materials, such as nickel titanium (NiTi), can also be examined to improve device benchmarking in the future. The FE models relied on the LHPM, where anatomical features such as the papillary muscles, the chordae and the heart valve were not modelled. These anatomical landmarks may complicate deployment and impact local stress during the cardiac cycle. The deployment process can be simulated over several heartbeats to thoroughly investigate the impact of the heart, predominantly during contraction.

The *in silico* models of healthy and HFpEF geometries were generated based on animal models and clinical measurements and validated against physiological strain measurements [33]. The implantation of the device was not part of the animal experiment, and therefore a theoretical evaluation through computational analysis was performed. As such, similar validation of the result could not be ascertained. Furthermore, it was impractical to validate the computational data by comparing it to the clinical results of previously implanted porcine models, as the implantations were carried out in fully grown pigs and the animal experiment did not include a comprehensive scan for an accurate comparison. Future research with a larger cohort and subtype can include device implantation in different configurations, as we considered for the optimization, to enable comparison between the clinical outcome and the numerical estimation. To fully appreciate the diversity in the population, twin cardiac models can be included in the analyses. These provide feasible anatomies, while not entirely subject-specific in scope [44], to represent different geometrical dimensions and functionality. To explore the effect on the human heart, our current findings, as well as selective cases, can be used for calibration and validation. The Living Heart Human Model [38,39], which provides a more precise and comprehensive representation of the human heart, can be used by employing the same methodologies to generate subject-specific and twin models as previously discussed.

In conclusion, the device was found to have a beneficial effect on heart function. There were no gross irregularities in stress magnitudes, supporting a basic safety profile. Altering the device's material properties and adapting its orientation can help improve heart function even more.

## Data Availability

Data available from the Dryad Digital Repository: https://doi.org/10.5061/dryad.m63xsj46m [45]. The data are provided in electronic supplementary material [46].
